# Disinfection1Read before the American Veterinary Medical Association, New York City, September, 1899.

**Published:** 1899-11

**Authors:** E. A. A. Grange

**Affiliations:** Detroit, Michigan


### DISINFECTION.[1]

By E. A. A. Grange, M.D., V.S.,
DETROIT, MICHIGAN.

If we had the time at our disposal it would be interesting to follow the history of disinfection from the bygone days of prehistoric times, through the dark ages of the earlier Christian era, down to the more enlightened period of our own day. In doing so we would find every page of that history bristling with important information as to how, when, and why certain agents should be used in cutting short the attack or arresting the spread of some malignant plague, and those inklings of truth which illuminate men's minds, enabling them to wander through fields of research, which a moment before were in utter darkness, stand out in bold relief, from period to period, and illustrate in a most emphatic manner the benefit one man's brain is to another in overcoming insurmountable difficulties in his efforts to reach the ideal.

Robert Boyle, as far back as 1676, is quoted as saying in an essay: "He that thoroughly understands the nature of ferments

[1] Read before the American Veterinary Medical Association, New York City, September, 1899.

and fermentation shall probably be much better able than he that ignores them to give a fair account of divers diseases (as well fevers as others) which will perhaps be never properly understood without an insight into the doctrine of fermentations," giving color to the importance attached to sanitary science by some, even in those early days ; but the history of our subject is teeming with such expressions, so I will not quote further, but believe I may truthfully say that disinfection has made more gigantic strides toward the goal of perfection during the past twenty years than it did in all the twenty centuries preceding them. Perhaps paramount in these strides we have the teaching of Sir Joseph Lister, closely followed by the work of the Committee on Disinfectants of the American Public Health Association appointed in 1884, but not divulging its conclusions until they were carefully weighed for three years, when in 1887 the final report was made. I would like to read that report, but time will not permit to even quote from it, so for information concerning it I must refer you to that classical treatise upon bacteriology by Sternberg, which I may add is an ornament to that science.

Running neck and neck with this work, we have the modern ways and means for testing the various disinfectants, when they are presented to us by the manufacturer, accurately described in the recently published *Laboratory Work in Bacteriology*, by Prof. F. G. Novy, of the Michigan University, while the notes of Dr. Young, Secretary of the Maine State Board of Health are most instructive to those in search of practical information on disinfection or disinfectants, all of which impress us with the idea that the closets containing the secrets of disinfection are being speedily unlocked by earnest men of science on both sides of the Atlantic. It has been my ardent desire in presenting this subject to do so in a practical manner, or, rather, as practical as I could make it. I was, however, balked lately in some ways through unforeseen circumstances, but have since conceived others which, I trust, may answer the purpose for the time being.

At this point let me say that while disinfectants and antiseptics go hand-in-hand, it may be well to draw a line between them ; and will add, that while the antiseptic may inhibit or prevent the growth of the germ of contagion for a certain time, disinfectants in the true sense of the term totally destroy all contagious matter, be it germ, spore, or something else, and the matter of testing the value of these agents has received much consideration from bacteriologists for some years past ; this work is at times rather mystifying, as apparently conclusions are not always the same. This, however, may be the result of losing track, as it were, of some of the conditions. I doubt if I can illustrate this better than by quoting the Secretary of the State Board of Health of Maine, to wit:

"1. The power of resistance of the same species of bacterium varies greatly under different conditions or when their source is different. For instance, Baer found that a freshly inoculated culture of the bacillus of diphtheria was destroyed with a 1 : 5000 solution of nitrate of silver, but that a twenty-four-hour culture required 1 : 1000 of the same agent to sterilize it in the same space of time.

"2. The media in which test bacteria exist influence strongly the action of disinfectants, the bacillus of tuberculosis dried upon threads or in aqueous suspension may be destroyed by mercuric chloride, but in fresh tuberculous sputum it cannot be trusted to sterilize it. As illustrative of the influence of media, Behring says that sporeless antiseptic bacilli in water are killed by corrosive sublimate 1 : 500,000; in bouillon by 1 : 40,000; but in blood-serum not with certainty by 1 : 2000.

"3. The temperature under which a disinfecting material acts influences very much the rapidity and the certainty of its action. Thus, anthrax spores which survived the action of a 5 per cent. solution of carbolic acid thirty-six days at ordinary room temperature were killed in from one to two hours at 131° F., and in three minutes at 167° F.

"4. In many experiments the inhibitory action of the agent has been mistaken for its germicides or disinfectant action."

To the above I may add that in accepting statements as to the merits of certain agents we must not lose sight of the fact that all disinfectants are antiseptics, though all antiseptics are not disinfectants any more than that all horses are quadrupeds, but all quadrupeds are not horses. And in applying these remedies to our patients, we must be guarded in condemning a medicine which does not react in the desired manner, as the resistant force or idiosyncrasy of the animal, temperature of the surroundings, dry or moist conditions of the part, etc., may, one or all, play a most important rôle in the *modus operandi* of the drug.

And now let me call your attention briefly to some few agents used in the work.

I am often asked if lime-wash is not a good thing to disinfect a stable, and as it is so extensively used, some investigation was carried on at the Michigan Agricultural College, when I had charge of the bacteriological work, by one of our students, under the direct supervision of Mr. Charles E. Marshall, then my assistant, now bacteriologist to the Experiment Station, during which he produced his graduating thesis on "The Action of Whitewash upon Bacteria." The conclusions arrived at after a number of experiments had been carefully carried through were that in some instances the whitewash seemed to prevent the growth of the vegetative forms of several germs, but did not prevent the growth of the spore forms of the bacillus anthracis.

Were I requested to name the best disinfectant for all purposes, I hardly know what I would say; but so much has been recently said and written in favor of formaldehyde, and adding this to my own experience with the agent, I lean toward it as coming as near the ideal as anything with which I am familiar. In support of what I have just said, let me offer the following compilations:

Dr. Charles Harrington, Instructor in Hygiene and Materia Medica in the Harvard Medical School, as the result of elaborate and repeated experimentation, says that as a surface disinfectant formaldehyde possesses a power "greater than any other known substance."

Dr. E. A. de Schweinitz, Ph.D., says: "As compared with other disinfectants, such as corrosive sublimate, carbolic acid, lysol, etc., formaldehyde solutions have the advantage of not being retarded in their action by albuminous matters, and of not injuring the articles to which they are applied."

Trillat has proved it possible to completely disinfect rooms and the furniture contained therein by the consumption during six hours of from four to six litres of methylic alcohol for each 300 cubic metres of space.

Dr. J. J. Kinyoun, of the United States Marine Service, confirms the statement of Trillat, and shows that none of the ordinary fabrics are injured by the gas. It is entirely capable of completely disinfecting curtains, carpets, clothing, bed-covering, and the minor forms of furniture. (H. C. Wood, M.D., LL.D., in the *University Medical Magazine*, June, 1897.)

F. C. J. Bird, in the *Pharmaceutical Journal*, tabulates the purposes for which formaldehyde has been employed, and the proportions recommended. A solution of 1 : 125,000 kills anthrax bacilli; 1 : 50,000 prevents the development of typhus bacilli, etc.; 1 : 32,000 preserves milk for several days; 1 : 25,000 forms a useful injection in leucorrhœa, etc.; 1 : 20,000 preserves wines, weak alcoholic liquids, and beer, also milk for several weeks; 1 : 4000 is recommended for moistening paper used as a cover for jams, etc.; 1 : 3200 for rinsing dairy vessels, etc.; 1 : 2500 destroys the most resistant microörganisms in one hour; 1 : 2000 for rinsing casks and vessels intended for liquids liable to fermentation; 1 : 500 for the irrigation of catheters, etc., and as a mouth wash; 1 : 250 to 1 : 200 as a general disinfectant solution for washing hands, instruments in surgery, spraying in sick rooms, and as a deodorant; 1 : 160 to 1 : 100 hardens microscopic tissues, which should be immersed for a considerable time to give the best results; 1 : 100 in lupus, psoriasis, and other skin diseases; 1 : 50 to 1 : 25 sterilizes surgical catgut, silk, etc., by steeping; 1 : 25 for quickly hardening and preserving microscopical sections (longer immersion in a weaker solution gives better results); 1 : 10 for hardening very firm tissues in pathological and histological work; 1 : 5 for hardening firm tissues in such work; 1 : 2.5 for hardening soft tissues for the same purpose.

It is claimed that a 0.4 per cent. solution will almost immediately and entirely deodorize feces (*University Medical Magazine*, vol. ix., No. 9, p. 608). Among the minor applications of such a powerful and convenient deodorant may be mentioned the treatment of that very annoying and stubborn condition—offensive perspiration, especially of the feet.

It was inevitable that a substance of such germicidal activity as formaldehyde should be employed, tentatively, at least, in the treatment of suppurating wounds, ulcers, etc. Dr. Alexander, quoted by Dr. Leech in the *Medical Chronicle* for December, 1896, uses the 40 per cent. solution in chancroids and chancre, applying locally, prompt healing following a single application. He finds a spray of 0.5 per cent. of the 40 per cent. solution valuable in hay-fever, and a spray of 1 per cent. of the same solution in whooping-cough.

In the University Hospital, as well as the Presbyterian Hospital, and in private practice, Prof. Willard, of Philadelphia, has used formaldehyde in all sorts of wounds, in carbuncles, and in various infective sores. For washing out and purifying an infected wound he employs a 2 per cent. solution; for a continuous local application or for free irrigation, a 0.25 per cent. solution; and while the effects upon suppuration and the general evidences of infection have been very pronounced, in no case has there been any local irritation. (Wood, *loc. cit.*)

The testimony of Dr. Alexander, favorable to the employment of formaldehyde solutions in cases of gonorrhœa, is reinforced by the experience of several other physicians.

In ophthalmic practice, in the treatment of corneal ulcers, follicular inflammations, trachoma, etc., successful results have been reported by a number of practitioners, among them Burnett, Davidson, and Stephenson.

According to an abstract in the Cincinnati *Lancet-Clinic*, November 7, 1896, of forty cases of ringworm of the scalp, hospital out-patients, treated by means of formaldehyde in 40 per cent. solution vigorously rubbed in with a brush or mop for ten minutes, only five required repeating; the remainder were cured by one treatment. Microscopical examinations were always made before commencing treatment, and the presence of the trichophyton demonstrated.

Formaldehyde solution has been employed quite extensively in the treatment of throat affections. Yatcouta (*Revue de Therapeutique*, April 15, 1897) asserts that in sixteen cases of acute laryngitis in which he employed inhalations of a 2 per cent. aqueous solution of formaldehyde a complete cure was affected in from seven to twenty-four hours. In three cases of acute coryza the condition disappeared in twenty-four hours after the use of three or four douches of a weak formaldehyde solution.

Dr. W. S. Alexander, of Oxford, Ohio, in a paper read before the Union District Medical Association and published in the Cincinnati *Lancet-Clinic*, expresses his enthusiastic approval of formaldehyde as a remedy for hay-fever. He employs a 0.5 per cent. solution of 40 per cent. formaldehyde, allowing the patient to inhale the fumes from a drachm vial. He claims that in this way he comes as near curing the most obstinate forms of catarrhal trouble as by all other means known, which suggest its use in influenza of horses, etc.

It is stated by Mr. Bird (quoted above) that meat, fish, etc., may be kept for several days during the hottest weather by placing them in a well-covered dish with a tuft of cotton-wool moistened with four to eight drops of formaldehyde solution. This antiseptic vapor does not communicate the slightest odor or taste to the meat, etc.

The property which formaldehyde possesses of preserving animal tissue seems to be due to a penetrating action whereby it readily combines with the protoplasm of the cells, checking not only fermentative changes, but changes of growth as well. "By repeatedly painting the ear of a rabbit with a concentrated solution (not stronger than 40 per cent.) in ten days the ear will fall off as smoothly as if cut off, and without bleeding. Quite similar is the effect upon the human epidermis. The application of this action of formaldehyde solution to surgery is obvious." (Alexander, *loc. cit.*)

The manifest interest in formaldehyde at the present time prompts me to place in your hands a graphic description of some 5000 experiments conducted in the Hygienic Laboratory of the University of Michigan. It speaks for itself.

A similar generator to the one before you was used in the work. Sheets saturated with a desired quantity of the fluid and suspended around the compartment answered the purposes of disinfection very nicely, although they may not be as convenient in all instances as a commercial generator.

My attention has been somewhat forcibly directed during the last couple of years to a practical disinfecting material developed by Dr. Charles T. McClintock as a convenient, effectual, and agreeable method of disinfecting the hands, instruments, wounds, or for destroying fleas on dogs, etc., I can hardly speak too highly of it, especially for destroying fleas on dogs; but to do it anything like justice in the time I have at my disposal, I must place in your hands a reprint containing the formula, in case you desire to make it yourselves, beside a description of numerous experiments made by competent persons for the purpose of verifying claims made as to the merits of the article. I also hand you a small cake of the soap, which will enable you to test its virtues if you so desire.

Mercurol is another new disinfectant, developed by Dr. Schwickerath, which I would like to talk about, but time will not permit, so will once more have to take advantage of the reprint and actual specimen.

In conclusion, I have to thank you very much for the patient consideration you have given my somewhat disconnected remarks, but if I have done nothing more I feel that I have shown you that in an ordinary half-hour one cannot get even beyond the threshold of disinfection, so I now leave the question in your hands.

---

Dr. Otto Noack, of Reading, Pa., was a visitor to the JOURNAL office in October, and spent the evening with the Keystone Veterinary Medical Association.

